# Food Acquisition, Preparation, and Consumption Practices in South Asia: A Scoping Review of Assessment Tools

**DOI:** 10.1016/j.advnut.2025.100518

**Published:** 2025-09-18

**Authors:** Sharvari Patwardhan, Morgan Boncyk, Rasmi Avula, Christine E Blake, Fahmida Akter, Jai K Das, Renuka Silva, Purnima Menon, Samuel Scott

**Affiliations:** 1 Nutrition Diets & Health Unit, International Food Policy Research Institute, New Delhi, India; 2 Arnold School of Public Health, University of South Carolina, Columbia, SC, United States; 3 BRAC James P Grant School of Public Health, BRAC University, Dhaka, Bangladesh; 4 Institute for Global Health and Development, Aga Khan University, Karachi, Pakistan; 5 Department of Nutrition and Dietetics, Wayamba University of Sri Lanka, Makandura, Gonawila, Sri Lanka; 6 Nutrition Diets & Health Unit, International Food Policy Research Institute, Washington, DC, United States

**Keywords:** food choice behaviors, food acquisition, food preparation, household consumption practices, South Asia, scoping review, assessment tools

## Abstract

Assessing behaviors related to food choice at individual and household levels is essential for improving household diets, but assessment tools are limited. We conducted a scoping review to identify gaps in existing assessment tools for food acquisition, preparation, and household consumption practices in South Asia, where diets are rapidly changing. We undertook systematic keyword searches of 3 databases (PubMed, Scopus, and Web of Science Core Collection) to identify studies assessing food acquisition, food preparation, and household consumption practices in Afghanistan, Bangladesh, Bhutan, India, Maldives, Nepal, Pakistan, and Sri Lanka, published in English between 2000 and April 2025. Two reviewers independently screened titles, abstracts, and full texts and extracted data on study characteristics and the assessment tools used to examine the food choice behaviors. Of 13,160 unique articles identified, 50 were included for synthesis. Food acquisition behaviors (e.g., what and how often food is purchased, changes in food purchases) were assessed by 26 studies, food preparation (e.g., cooking habits, intrahousehold distribution of responsibilities, preparation methods) by 9 studies, and household consumption practices (e.g., timing, snacking, meal skipping, eating away from the home) by 30 studies. Most studies used quantitative methods (*n* = 34), some used qualitative methods (*n* = 13), and few used mixed methods (*n* = 3). Likert scales and semistructured interviews were the most used tools for quantitative and qualitative assessments, respectively. Across the 50 studies, 40 different tools were used to assess food-related behaviors, and only 14 studies claimed to be using validated tools. Few studies included a full tool in the text or supplemental material (*n* = 23). Currently, there is little alignment on how to assess food choice behaviors in South Asia, highlighting the need for a contextually adaptable repository of tools. Adapting and validating existing tools, rather than creating new ones, could improve efficiency, continuity, and comparability.

This study was registered at Open Science Framework Registries as https://doi.org/10.17605/OSF.IO/5GPEF.

**Statement of Significance**

Although there is evidence on the assessment of the external food environment, the assessment of individual and household food choice behaviors within the personal food environment has received less attention. This scoping review addresses this evidence gap by synthesizing how food acquisition, preparation, and household consumption practices have been assessed across South Asia, providing a region-specific overview of existing tools and identifying key methodological gaps in the measurement of food choice behaviors.

## Introduction

Healthy diets play an important role in preventing all forms of malnutrition and diet-related noncommunicable diseases [1]. Individual food choices that shape dietary patterns are important for achieving sustainable healthy diets. In South Asia, where healthy diets are challenged by poverty, high food prices relative to income, rapidly evolving food environments, and availability and access constraints [1,2], solutions require an understanding of the external food environment along with an examination of food choice behaviors at household and individual levels [[3], [4], [5]]. A group of nutrition experts who convened in South Asia recently noted the importance of building evidence on drivers of food choice and on tools for monitoring and assessing food choice behaviors [6].

Food choice is not limited to food consumption alone but rather encompasses processes by which individuals decide what, how, and why to acquire, prepare, allocate, store, consume, and dispose of food [7,8]. These processes involve a series of food-related decisions and behaviors within the context of specific food environments, which can be physical or digital spaces [4,5]. Food choice is intertwined with expressions of identity, preferences, and sociocultural values that ultimately shape dietary intake and health outcomes. Such “drivers” of food choice span individual and household levels and are influenced by broader community and macrofactors [7,8].

Food acquisition refers to what people acquire and how and where they acquire it, whereas food preparation refers to actions performed to transform food from raw or processed (partially or fully) ingredients to a consumable form in the household [7]. Household food consumption practices are described according to their patterning (e.g., regularity, skipping, or timing), format (e.g., sequence of consumption of food groups), and context (e.g., family meals, engagement in co-occurring behaviors such as watching television) [7].

Limited knowledge of food choice behaviors and their linkages stems partly from a dearth of valid and reliable tools to assess these behaviors [9]. Recent methodological advancements to assess food environments in low- and middle-income countries emphasize the external food environment (availability, prices, vendors and product properties, marketing, and regulation) [[3], [4], [5]]. However, assessment of individual and household food choice behaviors within the personal food environment has received less attention [9]. Understanding and assessing these food choice behaviors and their effect on diets and health is important for developing policies and programs to improve the healthfulness of diets.

This scoping review of methods aimed to understand how food acquisition, food preparation, and household consumption practices were assessed in South Asia. A secondary aim was to understand the types of food choice drivers being studied.

## Methods

### Data search, screening, and inclusion criteria

This review was registered in Open Science Framework Registries (https://doi.org/10.17605/OSF.IO/5GPEF) and followed PRISMA guidelines. Referring to the drivers of food choice and food choice behaviors constructs [8], we conducted an iterative scoping exercise to identify, test, and refine the appropriate search strings for the 3 food choice behaviors of interest—food acquisition, preparation, and household consumption practices (Supplemental Table 1). The emphasis was on research that assessed how people eat rather than what people eat. Hence, we did not include assessments of dietary intake in our understanding of food choice behaviors. We did not include intrahousehold food allocation in the review as there is an existing systematic review on this behavior [10].

Two authors (SP and MB) performed the searches in April 2025 in PubMed, Scopus, and Web of Science Core Collection, filtered for publications in English published since 2000. The keyword search in the 3 databases was restricted to the title and abstract. We conducted a separate search for each of the 3 food choice behaviors. We pooled the results for all 3 behaviors and from all 3 databases and removed duplicates prior to screening. We imported search results into Rayyan, a web tool designed for systematic literature reviews [11], to aid in identifying duplicates and carry out the screening process.

Two reviewers (SP and MB) independently screened titles, abstracts, and full texts, and a third reviewer (SS, RA, or CEB) resolved any disagreements. All reviewers met weekly during the screening process to ensure consensus and discussed any uncertainties until resolution was achieved.

Articles were included if they were based in a country in South Asia (Afghanistan, Bangladesh, Bhutan, India, Maldives, Nepal, Pakistan, or Sri Lanka), assessed food acquisition, food preparation, or household consumption practices and had ≥1 driver of food choice at the individual or household level, described the tool used to assess the food choice behavior(s), were peer reviewed, published in English language from January 2000 to April 2025, and involved a generally healthy population (not in hospital, without conditions requiring ongoing medical attention).

### Data extraction and analysis

Two authors (SP and MB) independently extracted data, and a third reviewer (SS, RA, or CEB) independently reviewed the extracted data to minimize potential bias and verify the accuracy of the extracted data. We extracted data on authors, title, study country, residence type (rural, urban, or periurban), study objectives, study population (age and sex), method of assessment (quantitative or qualitative), study design, behaviors and drivers assessed, tools used to assess the behavior(s), availability of the assessment tool, and whether the assessment tool was validated.

We grouped data according to food choice behavior [7]. We grouped food choice drivers as intrapersonal drivers, sociocultural drivers, personal food environments, material assets and resources, or person-state drivers [7]. We further categorized quantitative assessment tools as self-administered or interviewer-administered questionnaires and qualitative tools as semistructured interviews, focus group discussions (FGDs), pile sorting, or photovoice. The availability of questionnaires or tools used to assess the behavior(s) was categorized as “descriptions of questions specified,” “few questions specified,” or “complete tool included.” We reviewed the article text and supplemental material to locate specific questions. We described quantitative tools as validated if authors stated they either used or validated a tool within the study context. Validity was not applicable for qualitative tools.

## Results

### Summary of evidence across food choice behaviors

**FIGURE 1:**
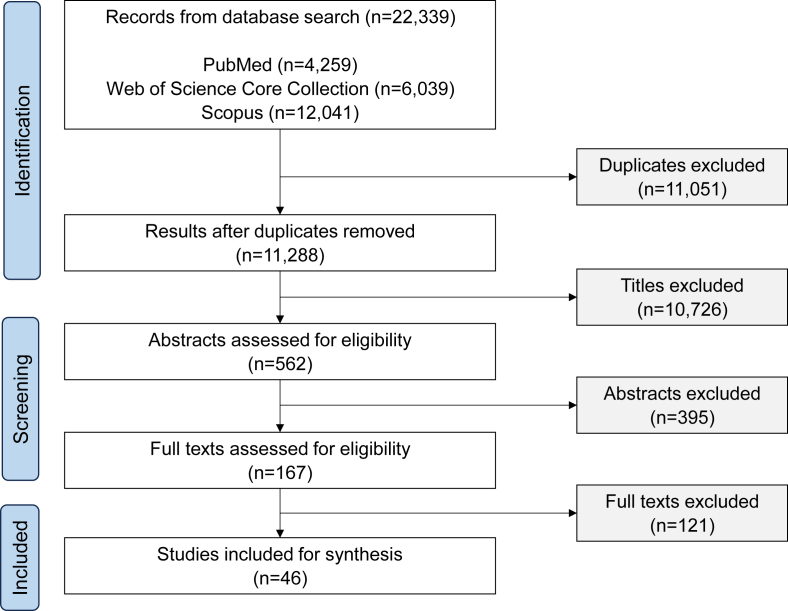
PRISMA-driven flowchart for selection of studies included in the review.

We identified a total of 25,406 articles. After removing duplicates, we screened 13,160 titles, 619 abstracts, and 186 full texts (Figure 1). A total of 50 studies met the inclusion criteria and were included for synthesis. Details on the study population, data collection method, food choice behaviors assessed, and domains of food choice drivers assessed in each of the 50 studies are shown in Supplemental Table 2.

Food acquisition behaviors were assessed by 26 studies, food preparation by 9 studies, and household consumption practices by 30 studies (Table 1). Most of the studies across all 3 behaviors were published in recent years, with the highest concentration between 2021 and 2025 (*n* = 22). In contrast, no studies were published between 2000 and 2005, indicating a growing interest in assessing these behaviors in South Asia in the past 2 decades. Most studies were from India (*n* = 37), and few were from Bangladesh (*n* = 5), Pakistan (*n* = 4), Nepal (*n* = 3), and Sri Lanka (*n* = 1). No studies were identified from Afghanistan, Bhutan, or Maldives, and no studies examined multiple countries. Over two-thirds of the studies occurred in an urban setting (*n* = 25), 3 were from a rural setting, 1 was from a periurban setting, and 7 did not specify a setting. Most studies included only adults (*n* = 30), whereas few included only adolescents (*n* = 8), younger children (*n* = 2), or had mixed age groups (*n* = 10). Most studies (*n* = 42) included both male and female participants.

**TABLE 1:**
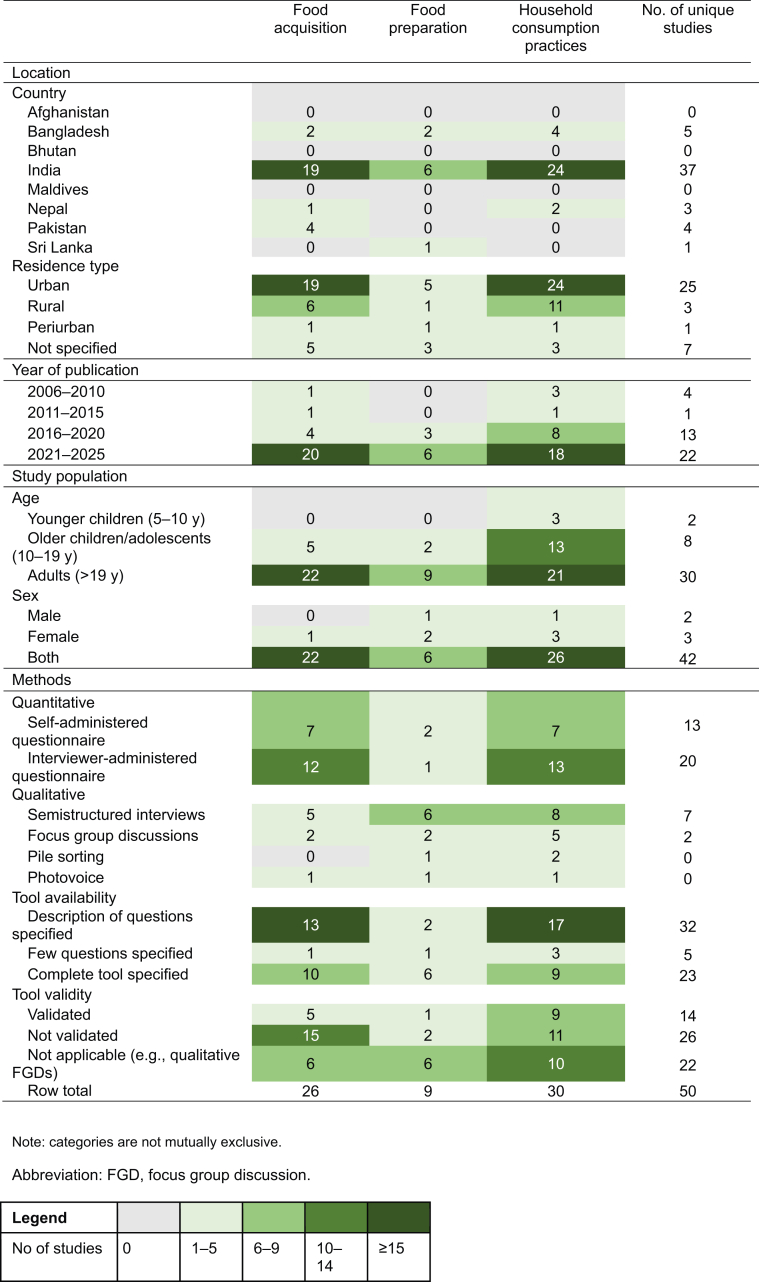
Summary of the number of studies included by food choice behavior.

Thirty-four studies used quantitative methods, 13 studies used qualitative methods, and 3 studies used mixed methods. Quantitative tools included questionnaires (self- and interviewer-administered) with various scales, and qualitative tools included semistructured interviews and FGDs. Across the 50 studies, 40 different tools were used; only 14 studies used validated tools as reported by the authors. A complete tool was provided in 23 studies, with the remaining studies only describing questions or providing a few questions.

### Tools used to assess food choice behaviors

#### Tools for assessment of food acquisition behaviors

Twenty-six studies described food acquisition behaviors in terms of frequency of purchases (e.g., frequency of buying outside food, buying from the school canteen) [[12], [13], [14]], purchase of specialty foods (e.g., supersized foods, halal food, and organic food) [[15], [16], [17], [18], [19], [20], [21], [22], [23], [24], [25], [26], [27], [28]], influence of the COVID-19 pandemic on food buying behavior (e.g., changes in food shopping behavior, market frequency) [[29], [30], [31], [32], [33]], intrahousehold acquisition practices (e.g., where and who acquires food) [34,35], and online purchasing (e.g., pattern of online food orders, consumption occasions for ordering online food) [36,37] (Table 2). Frequency of food purchases was assessed using both quantitative (questionnaires) [12,14] and qualitative (FGDs) [13] approaches. Most studies assessing purchase behavior for specialty foods (in terms of purchase frequency, purchase patterns, and purchase preferences) used quantitative tools. Likert scales [[15], [16], [17],^19^,^20^,^24^,^26^,^33^] were most commonly used by interviewer-administered surveys [18,21,27,33] and online questionnaires [25], followed by qualitative tools such as semistructured interviews [23,28] including open-ended questions [22]. Changes in food acquisition behaviors during the COVID-19 pandemic were assessed primarily using quantitative self-administered [[29], [30], [31]] and interviewer-driven questionnaires [33], whereas only 1 study conducted qualitative FGDs [32]. Studies examined intrahousehold food acquisition practices using qualitative interviews [[34], [35], [36]], which were supplemented with photovoice [34] and quantitative surveys [35] administered via phone and mail [37].

**TABLE 2:** Summary of tools used to assess food acquisition behaviors.

Assessment tool	Population and context	Description of behaviors assessed	Description of scale/questions
Frequency of food purchases			
Qualitative: focus group discussions	Adolescents and their caregivers living in urban slums (urban, India)	Frequency of buying outside food	How frequently do your family members give you money to buy outside food? What foods do you buy with the money given by the family? [13]
Quantitative: survey (Interviewer-administered questionnaire)	Low- and middle-income households (urban and rural, India)	Purchasing behaviors	The questionnaire collected information about consumers’ purchase and physical access to various food products and perceptions on the promotional aspect of food [14].
Quantitative: survey (Self-administered questionnaire)	School-going adolescents (urban, India)	Frequency of buying from the school canteen	Do you eat at the school canteen or buy foods/beverages at school? If yes, then how often…? Why do you buy these foods? Response options included taste, price, availability, and convenience [12].^1^
Purchasing behavior of specific foods			
Qualitative: interviews (semistructured interviews)	Consumers of supersized foods (urban, Pakistan)	Supersized food purchase behavior	When was the last time they ordered supersized food/drink? What causes consumers to purchase supersized food? Does larger package or supersized food within an assortment of product sizes reflect status? [22]
	Consumers of western-imported foods (urban, Pakistan)	Western-imported foods purchase behavior	Specific questions not mentioned [23].
	Consumers of western-imported foods (urban, Pakistan)	Buying behavior toward edible oil and vanaspati ghee	Preference of respondent takes value 1 if oil is preferred and vanaspati ghee otherwise. Preference of oil assessed against health concern, or due to taste/aroma (1 if Yes and No otherwise) [28].
Quantitative: survey (interviewer-administered questionnaire)	Adults (urban, India)	Frequency of purchasing probiotics	Questions helped to access acceptance, brand awareness, and willingness to purchase probiotic products [18].
	Adolescents (urban, India)	Fast food buying behavior	Factors influencing buying behavior toward fast food were recorded on a 3-point Likert scale (3 = most influencing factor, 2 = moderately influencing factor, and 1 = least influencing factor) [19].^1^
	Consumers (urban, Bangladesh)	Halal food purchase behavior	How many times would you say you have purchased halal food per month? How long have you been buying halal foods? [20]
	Consumers (urban, India)	Meat purchase patterns	Information collected on mode of purchase (from shop, slaughter in home or front of eyes, frozen/supermarket) and preferred product type [21].
	Consumers (urban, India)	Purchase behavior for health and wellness food products	How often do you purchase organically grown produce or other organic food products? [17]
	Consumers of organic food (urban, Nepal)	Purchase frequency of organic foods	Factors affecting buying behavior were recorded on a 5-point Likert scale (1 = not at all important and 5 = extremely important) [24].
	Experts in the food industry (India)	Purchase process of organic foods	The questionnaire involved a mixture of structured and semistructured questions. Respondents evaluated the importance of 19 variables in the consumers’ purchase process on a 1–5 scale [27].
Quantitative: survey (self-administered questionnaire)	Consumers representative of households (urban and rural, India)	Nonpackaged nonbranded rice purchase behavior	Questions about frequency of purchase, quantity purchased, source of purchase, and mode of purchase [25].
	Young consumers/students (urban and rural, India)	Purchase frequency of organic foods	How often have you shopped for organic foods in the last 3 mo (1 = “never”; 5 = “at every opportunity”)? How many organic foods have you bought over the last 3 mo (1 = “none at all”; 5 = “a great deal”)? [15] 1
	Adult respondents interested in organic foods (urban, India)	Purchase frequency of organic foods	Responses to the statement “Occasionally, I purchase organic foods” were recorded on a 5-point Likert scale, from agree to disagree [16].
	Young consumers/students (urban and rural, India)	Purchase frequency of organic foods	All constructs about factors influencing organic food purchases were measured on a 5-point Likert scale, ranging from 1 for “Strongly disagree” to 5 for “Strongly agree” [26].^1^
Changes in food acquisition behavior due to the COVID-19 pandemic			
Qualitative: interviews (semistructured interviews)	Female food gatekeepers/ those responsible for feeding their family members, COVID-19 pandemic (urban, India)	Food shopping behavior	Can you explain your food shopping experience before and during lockdown? From which sources do you usually procure food on a regular basis (before and during lockdown)? [32]
Quantitative: survey (self-administered questionnaire)	Adults, COVID-19 lockdown (India)	Market frequency	Information collected on the frequency of going to market during the lockdown period [29].
	Adults, COVID-19 pandemic (urban and rural, India)	Food buying behavior	How much has COVID-19 reduced buying frequency of food purchases? Has COVID-19 changed buying behavior toward online purchases (1 if people changed buying behavior, and 0 otherwise)? [30]
	Consumers, COVID-19 lockdown (India)	Meat buying behavior	Questionnaire collected information on meat procurement source, type of meat/meat product purchased, meat available even during the lockdown period, and the quantity of meat procured during COVID-19 lockdown compared with the normal situation [31].^1^
Quantitative: survey (interviewer-administered questionnaire)	Adults, COVID-19 pandemic (Bangladesh)	Food buying behavior	Responses recorded on a 5-point Likert scale (ranging from “never” to “always”) regarding various aspects such as: purchasing food from the supermarket, purchasing food from the grocery store, purchasing food from the local market, purchasing food from online grocery shopping sites, and ordering food from online food delivery platforms [33].
Intrahousehold food acquisition practices			
Qualitative: interviews (semistructured interviews)	Periurban households (periurban, India)	Food acquisition practices	In-depth interviews about current food acquisition practices and intrahousehold food acquisition [34].
	Low- and middle-income households (urban, India)	Where and who acquire food	Key informants (food vendors) were interviewed to explore food purchasing behavior of the households [35].
Qualitative: photovoice (participant photographs)	Periurban households (periurban, India)	Food acquisition practices	Participants photographed their food acquisition practices over a 3-d period. With these, photoelicitation follow-up interviews were conducted with participants [34].
Quantitative: survey (interviewer-administered questionnaire)	Low- and middle-income households (urban, India)	Where and who acquire food	The structured survey schedule focused on the following: *1*) usual food acquisition in the household, *2*) household decision making on food purchases, *3*) the usual food purchaser, and *4*) the location of where food purchases were made [35].
Online food purchases			
Qualitative: interviews (semistructured interviews)	Adults, COVID-19 lockdown (India)	Pattern of online food orders	A subset of participants who were found to be food addicts based on the Yale Food Addiction Scale were asked: During this lockdown phase, have you ordered ready-to-eat (cooked) food online? If yes, how many times (Yes/No/Other) [36]
Quantitative: survey (self-administered questionnaire)	Adults, COVID-19 lockdown (India)	Pattern of online food orders	Yale Food Addiction Scale used to assess whether respondents were food addicts. The questionnaire asked if participants’ eating behavior caused significant distress and also whether they experienced significant problems in being able to function effectively due to their food or eating habits. Responses recorded on a 5-point Likert scale [36].
	Online food delivery customers (urban, India)	Recurring consumption occasions for ordering online food and factors impacting the same	The questionnaire had subsections including demographic profile, consumption and spending pattern, factors affecting online food ordering, and overall satisfaction [37].
Quantitative: survey (interviewer-administered questionnaire)	Online food delivery customers (urban, India)	Recurring consumption occasions for ordering online food and factors impacting the same	The questionnaire had subsections including demographic profile, consumption and spending pattern, factors affecting online food ordering, and overall satisfaction [37].

1 Validation of the tool is reported in the Methods section of the article.

Likert scales were the most commonly used quantitative tools, and semistructured interviews were the most commonly used qualitative tools. Among the 5 studies that used Likert scales [16,19,24,26,33], there was limited convergence in terms of tool design. Although most tools assessed urban settings, primarily in India [16,19,26], the specific constructs varied. For example, 2 studies used Likert scales as tools to assess factors influencing buying behavior, but 1 study used a 3-point scale (least to most influencing) and was validated [19], whereas the other used a 5-point scale (not at all to extremely important) and was not validated [24].

Overall, although most tools captured aspects such as the frequency, mode, and motivations for food purchases, there was divergence in both design and applicability. This was particularly evident in the type of food examined. Although a few tools focused on general food acquisition behaviors, the majority assessed the acquisition of specific food types (e.g., organic, fast food, halal), limiting the generalizability and replicability of tools used. Additionally, as most of the tools were used in urban settings, efforts are needed to improve tool applicability to rural and periurban contexts.

Of the 14 studies that quantitatively assessed food acquisition behaviors, 5 reported the use of validated instruments [12,15,19,26,31], with only 1 employing an interviewer-administered questionnaire. This finding underscores the limited availability of validated tools for measuring food acquisition behaviors. To advance research in this area, future studies should prioritize the adaptation and validation of existing quantitative Likert scales and semistructured interview protocols. The integration of mixed-method approaches could also enhance the depth and scope of food acquisition research across diverse settings.

#### Tools for assessment of food preparation behaviors

Nine studies [13,29,[32], [33], [34],^36^,[38], [39], [40]] described assessment of food preparation behaviors, including intrahousehold distribution of food preparation responsibilities [34,40] and cooking habits (e.g., the type of food typically prepared, time spent in the kitchen) [13,29,32,33,38,39] (Table 3). Intrahousehold distribution of food preparation responsibilities was assessed using qualitative tools such as semistructured interviews [34,40] and photovoice [34]. Two studies conducted semistructured interviews on general intrahousehold food preparation [34] and how food preparation and cooking responsibilities differ between household members [40]. Cooking habits were examined using qualitative interviews [32,36,39], FGDs [13,39], pile sorting [38], and quantitative surveys [29,33].

**TABLE 3:** Summary of tools used to assess food preparation behaviors.

Assessment tool	Population and context	Description of behaviors assessed	Description of scale/questions
Intrahousehold distribution of food preparation responsibilities			
Qualitative: interviews (semistructured interviews)	Households (urban and rural, Sri Lanka)	Preparation and cooking roles	How do food preparation responsibilities and cooking responsibilities differ between household members? [40]
	Periurban households (periurban, India)	Intrahousehold food preparation	In-depth interviews about intrahousehold food preparation [34].
Qualitative: photovoice (participant photographs)	Periurban households (periurban, India)	Intrahousehold food preparation	Participants photographed their food preparation practices over a 3-d period. With these, photoelicitation follow-up interviews were conducted with participants [34].
Cooking habits			
Qualitative (semistructured interviews)	Resident students in a public university (urban, Bangladesh)	Cooking habits	Please say something about your food selection. What type of food do you eat? How and when do you eat it? [39]
	Female food gatekeepers/ those responsible for feeding their family members during COVID-19 pandemic (urban, India)	Meal preparation, household cooking, and kitchen experimentation during COVID-19	Can you describe your meal planning behavior before and during lockdown? [32]
	Young adult women (urban, India)	Preparation methods	Is that how you typically prepare [name a specific food or drink]? [If “Yes”], why do you prepare it this way? [If “No”], how else do you prepare it? Why? [38]
	Adults, COVID-19 lockdown (India)	Tried to cook to satisfy cravings	A subset of participants who were found to be food addicts based on the Yale Food Addiction Scale were asked whether they cooked food at home to satisfy cravings (yes/no/other) [36].
Qualitative (FGDs)	Resident students in a public university (urban, Bangladesh	Cooking habits	Please say something about your food selection. What type of food do you eat? How and when do you eat it? [39]
	Adolescents and their caregivers living in urban slums (urban, India)	Frequency of certain food preparation	How often does your mom/ caregiver prepare your favorite dish? [13]
Qualitative (pile sorting exercises)	Young adult women (urban, India)	Preparation methods	Respondents were asked to sort a list of 12 a priori hypothesized drivers into categories of always, sometimes, and never influencing food choice [38].
Quantitative: survey (self-administered questionnaire)	Adults, COVID-19 lockdown (India)	Time spent in the kitchen	Are you spending much time in the kitchen than usual? In the lockdown, your cooking skills and regularity improved? (no/not preparing foods/Yes) [29]
	Adults, COVID-19 lockdown (India)	Tried to cook to satisfy cravings	Yale Food Addiction Scale used to assess whether respondents were food addicts. The questionnaire asked if participants’ eating behavior caused significant distress and also whether they experienced significant problems in being able to function effectively due to their food or eating habits. Responses recorded on a 5-point Likert scale [36].^1^
Quantitative: survey (interviewer-administered questionnaire)	Adults, COVID-19 pandemic (Bangladesh)	Meal preparation practices	Responses recorded on a 5-point Likert scale (ranging from “never” to “always”) regarding various aspects such as: trying new recipes, preparing family meals more frequently [33].

Abbreviation: FGD, focus group discussion.

1 Validation of the tool is reported in the methods of the manuscript.

Most studies assessing food preparation behaviors used qualitative tools such as semistructured interviews [32,34,36,[38], [39], [40]]. However, these tools varied considerably in terms of the scope of preparation behavior assessed, tool design, and contextual focus. For instance, of the 2 studies that examined household food preparation, 1 investigated preparation practices [34], whereas the other focused on the division of responsibilities [40], highlighting limited convergence in what was measured. Furthermore, most tools were administered in urban settings, which limits their applicability and replicability to rural and periurban populations.

Quantitative self-administered questionnaires were implemented by only 2 studies [29,36], each assessing distinct aspects of preparation behavior—1 examining time spent in the kitchen [29] and the other examining cooking to satisfy cravings [36]. Although both surveys employed structured response scales, there was little consistency in the design of these scales, with varied response categories, which limits comparability.

Only 1 of the 3 studies that quantitatively assessed food preparation behavior reported using a validated tool. This tool, based on the Yale Food Addiction Scale, measured food-addictive behaviors among adults and their influence on cooking during the COVID-19 lockdown [36]. Although validated, its scope is highly specific and therefore not generalizable to broader food preparation behaviors or diverse population groups.

In summary, current tools assessing food preparation behaviors show limited convergence in scope, design, and validation. Most focus on specific behaviors or populations and are concentrated in urban settings, limiting their generalizability. Future research should aim to broaden the scope of both qualitative and quantitative tools to capture a wider range of food preparation behaviors. Efforts are also needed to develop more standardized qualitative protocols and to validate quantitative instruments to enable cross-context comparisons.

#### Tools for assessment of household consumption practices

Thirty studies described household consumption practices, including intrahousehold food distribution and consumption [32,35,[41], [42], [43], [44]], foods consumed during various eating occasions [14,38], eating habits [33,34,[45], [46], [47], [48], [49], [50], [51]], eating behaviors [39,52], snack consumption patterns [12,13,[53], [54], [55], [56], [57]], and eating out behavior [[58], [59], [60], [61]] (Table 4).

**TABLE 4:** Summary of tools used to assess household consumption practices.

Assessment tool	Population and context	Description of behaviors assessed	Description of scale/questions
Intrahousehold food allocation practices			
Qualitative (semistructured interviews)	Low- and middle-income households (urban, India)	Food distribution patterns	In-depth interviews about intrahousehold food distribution patterns [35].
	Adolescent females from low-income households (urban and rural, Bangladesh)	Food allocation	Specific interview guidelines not mentioned [43].
	Female food gatekeepers/those responsible for feeding their family members), COVID-19 pandemic (urban, India)	Family mealtime	Can you tell me about your social eating dynamics (i.e., family mealtime) before and during lockdown? [32]
Qualitative (FGDs)	Families in the community (rural, India)	Eating order	Who eats first, and who eats last? What happens when the food is finished and the woman of the household has not yet eaten? Does she go back and cook more food? How else might she get food? [41]
Qualitative (pile sorting exercises)	Families in the community (rural, India)	Eating order	At the group and household level, flash cards were used to guide the facilitator in general discussion points [41].
Qualitative (free listing exercises)	Adolescent females from low-income households (urban and rural, Bangladesh)	Food allocation	Free listing exercises conducted to identify foods eaten during meals and eating patterns [43].
Quantitative: survey (interviewer-administered questionnaire)	Children and their parents from educationally backward areas (rural, India)	Whether girls eat after boys in the household	Parents asked to what extent they agreed with statements reflecting gender roles and attitudes. Responses collected on a 3-point Likert scale (agree/agree to an extent/disagree) [42].
	Low- and middle-income households (urban, India)	Food distribution patterns	Questions on intrahousehold food distribution patterns [35].
	Adults highly involved in decision making in the family (urban and rural, India)	Urban/rural differences in household food consumption	In which form do you usually buy wheat? Frequency of consumption of selected convenience food products; media that influences food products. Importance of 9 food choice motives scored between 1 and 7 [44].
Foods consumed during various eating occasions			
Qualitative (semistructured interviews)	Young adult women (urban, India)	Foods consumed during different eating occasions	Is this what you typically eat at this time of day? [If “No”], how is it different? [38]
Qualitative (FGDs)	Low- and middle-income households (urban and rural, India)	Frequency of eating occasions	Statements collated relating to food quality attributes for each eating occasion [14].
Qualitative (pile sorting exercises)	Young adult women (urban, India)	Foods consumed during different eating occasions	Respondents asked to sort a list of 12 a priori hypothesized drivers as always/sometimes/never influencing food choice [38].
Quantitative: survey (interviewer-administered questionnaire)	Low- and middle-income households (urban and rural, India)	Frequency of eating occasions	Respondents were asked to evaluate predefined statements relating to food quality attributes for each eating occasion (i.e., breakfast, morning snacks, lunch, afternoon snacks, and dinner) using a 5-point importance scale. Information regarding dishes consumed during daily eating occasions and the corresponding frequency of consumption of each dish was collected [14].
Eating habits			
Qualitative: interview (semistructured interviews)	Periurban households (periurban, India)	Eating practices	In-depth interviews about intrahousehold food consumption practices [34].
	Adolescents (urban and rural, Bangladesh)	Eating habits	Information on eating habits including meal type, meal frequency, eating breakfast, skipping meals, reasons for skipping meals, and water intake was recorded from participants [50].
Qualitative: photovoice (participant photographs)	Periurban households (periurban, India)	Eating practice	Participants photographed their food environment and consumption practices over a 3-d period. With these, photoelicitation follow-up interviews were conducted with participants [34].
Quantitative: survey (interviewer-administered questionnaire)	Adults, COVID-19 pandemic (Bangladesh)	Eating practice	Responses recorded on a 5-point Likert scale (ranging from “never” to “always”) regarding various aspects such as: having meals separately (away from family) more frequently than before, having family meals more frequently, outdoor dining, social dining, food takeaways, and snacking between meals [33].
	Young adults (urban, India)	Irregularity in eating habits	Participants were asked whether they regularly consume breakfast/lunch/evening food and the reasons for any irregularity in food habit [45].
	Adolescents of upper socioeconomic status in public schools (urban, India)	Meal skipping	Questionnaire included sections on demographic data, dietary habits, and exercise pattern [46].^1^
	Younger children (urban, Nepal)	Eating habits	Junk food consumption was measured by observing if any or all types of junk food were consumed ≥3 times in the last 1 wk. Assessment of environmental factors such as conventional shop near home (yes/no), type of food at school (homemade/buy from shop/provided by school), food after school (homemade/buy from shop), money to buy food (never/sometime/most of the time/everyday), taking child shopping (never/sometime/most of the time/everyday) [49].^1^
	Adolescents (urban and rural, India)	Eating habits	Original variable recoded into “frequency of consumption of aerated drinks” with response: yes (for daily, weekly, and occasional consumption); and no (never). The predictors examined included behavioral variables (such as eating fried food and watching television) [51].
Quantitative: survey (self-administered questionnaire)	University students (urban, Nepal)	Eating practice and food choice	Food Choice Questionnaire contained 20 items designed to assess the importance of 9 factors. Each item scored on a 4-point importance scale. Eating practice was based on a scale of 2 (“1 = good eating practice,” and “0 = otherwise”) [47].^1^
	Adolescent school children (urban, India)	Eating habits	Frequency of major meals/d (1–2/3/>3 times), frequency of snacking (≤3/4/>4 times), history of skipping meals (never/sometimes/often), and history of eating outside home (never/sometimes/often) were recorded. Overall eating habits were determined by scoring the relatively poorer eating habits (based on inappropriate frequency of intake, skipping meals, and eating fast foods) [48].^1^
Eating behaviors			
Qualitative (semistructured interviews)	Resident students in a public University (urban, Bangladesh)	Eating behaviors	What/how/why/where do you eat? Who serves you and how? What affects your eating and how? Please say something about your food selection. What type of food do you eat? Please discuss elaborately (when, how, why, and why not?) [39].
Qualitative (FGDs)	Resident students in a public university (urban, Bangladesh)	Eating behaviors	How and what do you eat? In your opinion, what aspects/issues/elements affect your eating in and around your university? Please discuss elaborately when, how, why, and why not? [39].
Quantitative: survey (self-administered questionnaire)	School-going children (India)	Eating behaviors	8 dimensions of eating style in children assessed (responsiveness to food, enjoyment of food, satiety responsiveness, slowness in eating, fussiness, emotional overeating, emotional undereating, and desire for drinks). A total of 35 questions were asked with 5 options (never/rarely/sometimes/often/always). Each question related to a particular dimension [52].^1^
Snack consumption patterns			
Qualitative (semistructured interviews)	Consumers of various age groups (urban and rural, India)	Frequency, reasons, and timing of snack consumption	The questionnaire included 126 questions for 25 food groups. Consumption pattern (including frequency of consumption) of routine meals and snack foods for the past month, their timing, habits related to skipping meals, and factors associated with choice of different snacks were assessed [55].
Qualitative (FGDs)	Adolescents and their caregivers living in urban slums (urban, India)	Fast food consumption	How frequently do your family members give you money to buy outside food? What foods do you buy from the money given by the family? How often do you eat with your family/other families/elsewhere in the community? How do your friends and family affect your choice of food? [13]
Quantitative: survey (interviewer-administered questionnaire)	Medical students (India)	Regularly eating fast foods	Questions regarding respondents’ food habits and the barriers that prevented them from maintaining healthy dietary habits [54].
	Adults (urban and rural, India)	Context for snack consumption	Multiple choice questions were designed to understand the preferences for activities done while eating snacks and the top reasons for choosing snacks. Participants selected 3 from a list of options and ranked them in the order of preference [57].
Quantitative: survey (self-administered questionnaire)	Preuniversity students (urban and rural, India)	Fast food consumption patterns	Participants answered 10 questions to assess their knowledge and practice of fast food consumption (1 point for the right answer and 0 for a wrong answer). Overall score ranged from 0 to 10 [53].^1^
	School-going adolescents (urban, India)	Context for snack consumption	In the last 7 d, how many days did you perform the following activities such as watching television while eating? The response options, ‘never’ to ‘always,” indicating <1/wk to 6–7 times/wk, were scored on a 5-point scale from 0 to 4. Higher scores indicated a higher frequency of indulging in a specific eating habit [12].^1^
	Adults, COVID-19 lockdown (urban, India)	Context for snack consumption	The total number of meals consumed before and during lockdown, whether participants consumed snacks at those meals, and reasons for change in snacking pattern during lockdown were assessed using a pre-structured list of beverages and snacks (to examine intake during and before lockdown) [56].
Eating out behavior			
Qualitative (FGDs)	Adults of various socioeconomic groups (urban, India)	Frequency of eating out	What is your opinion about eating out? Probe questions: Do they have more or less fat, sugar, and salt? What do you think are the reasons for fast-food preference and dining out? How frequently do you eat out? [59]
Quantitative: survey (interviewer-administered questionnaire)	Children and their parents (urban and rural, India)	Frequency of eating out	The questionnaire enquired about preferences of eating out and if participants were affected by/changed their buying behavior in light of the information they interpreted from food labels. Another section included perceived concerns about television advertisements for children’s food [60].^1^
	Consumers (urban, India)	Frequency of eating out	Questionnaire included questions related to the frequency of eating out in a month, preference of cuisine between Indian or Chinese, vegetarian and nonvegetarian, spending per visits, and advertisement; recorded on a nominal scale [58].
Quantitative: survey (self-administered questionnaire)	Street food consumers (urban, India)	Eating food sold by street vendors	Word of Mouth scale, Food Neophobia scale, and Intention to Consume scale used. Responses to statements were recorded on a 5-point Likert scale (extremely unlikely to extremely likely) [61].

Abbreviation: FGD, focus group discussion.

1 Validation of tool is reported in the methods of the manuscript.

Semistructured interviews [32,35,43], FGDs [41], and systematic data collection methods such as pile sorting and free listing exercises [41,43] were the most common approaches to assessing intrahousehold food distribution and consumption, followed by quantitative Likert scales [42] and importance scores [44]. A combination of qualitative interviews [38], pile sorting [38], FGDs [14], and quantitative survey tools [14] were used to examine foods consumed during various eating occasions. For instance, in the pile sorting exercises, respondents were requested to sort a list of a priori hypothesized drivers into piles of always/sometimes/never influencing food choice [38]. Eating habits and behaviors were assessed primarily using quantitative tools [33,[45], [46], [47], [48], [49],^51^,^52^]; some studies used qualitative interviews [39,50], photovoice [34], and FGDs [39]. A higher proportion of studies assessing snack consumption patterns and eating out behaviors used quantitative tools [12,53,54,[56], [57], [58],^60^,^61^] followed by FGDs [13,59] and qualitative interviews [55]. FGDs enquired about the frequency of giving adolescents money to purchase food outside the home [13] and the frequency and rationale for eating outside the home [59].

A combination of quantitative and qualitative tools was used to assess a range of household consumption practices. However, there was limited convergence across these tools in terms of design, constructs measured, and contextual applicability. For instance, whereas 3 studies used quantitative methods to examine the context of snack consumption, their tool designs varied considerably. One study used multiple-choice questions to explore preferences and activities associated with snacking, another used a validated response scale to measure frequency of performing certain activities while eating, and a third study assessed whether snacks were consumed during meals using a structured checklist.

Quantitative questionnaires followed by semi-structured interviews and FGDs were the most commonly used tools. Even within quantitative surveys, tool designs varied considerably—some used Likert-type scales to assess frequency, importance, or agreement, whereas others relied on open-ended questions or categorical response formats. Such variations in tool design can influence how consumption practices are interpreted, limiting the generalizability of findings.

Nine of the 20 studies that quantitatively assessed household food consumption practices reported using validated tools. Although the methodological diversity provides a broad repository of tools for future researchers, there remains a pressing need for greater standardization and validation. Moreover, there were fewer tools that included younger children while assessing consumption practices, pointing to a critical gap in research. Future work should prioritize the adaptation of existing tools to maintain consistency in school design while adapting the tools to include younger populations and diverse contexts.

#### Tools for assessment of behaviors during the COVID-19 pandemic

Changes in behaviors during the COVID-19 pandemic were examined by 7 studies [[29], [30], [31], [32], [33],^36^,^56^]. A combination of qualitative and quantitative tools was used, similar to the broader methodological approaches used in nonpandemic contexts. However, many tools were adapted to include temporal comparisons (e.g., before compared with during lockdown) [[30], [31], [32]], assess behaviors such as online food purchasing [36], and explore changes in meal preparation [33] and snacking patterns [56] resulting from movement restrictions. For instance, of the 2 studies assessing online food ordering behavior, 1 study explored frequency of ordering ready-to-eat food online during the COVID-19 pandemic using semistructured interviews [36], whereas the other study explored consumption occasions for ordering online food in a non-pandemic context using an interviewer-administered survey [37]. The structural formats of the tools remained consistent, often employing Likert scales or structured response options, but the content was contextually modified to reflect the unique constraints and experiences of the pandemic period. These adaptations underscore the importance of developing flexible yet validated tools capable of capturing food choice behaviors across different experiences.

## Discussion

### Summary of findings

This review of studies from South Asia examined tools used to assess 3 food choice behaviors and showed that most evidence exists for household consumption practices and food acquisition behaviors, with few studies assessing food preparation behaviors. Most studies were conducted in India, and two-thirds of them assessed food-related behaviors among only adults. Most studies used quantitative methods. Likert scales were the most widely used quantitative tools, whereas semistructured interviews were the most common qualitative tools. Validated tools were used in 14 of the 34 quantitative studies. However, notably, none of the studies adapted a previously validated tool to fit their study context. This lack of contextual adaptation of already validated tools combined with large heterogeneity between studies in terms of methods and tools used to assess food choice behaviors limits the comparability of findings across studies. This review thus highlights the need for greater harmonization and validation of existing assessment tools and provides a synthesis of instruments that may inform and strengthen future research efforts. The insights presented in this review can serve as a foundation to guide the design and implementation of subsequent studies assessing food acquisition, preparation, and household consumption practices.

### Insights on methods and tools

Several insights related to methods and tools for assessment of food choice behavior emerged from this review. First, food choice behaviors are complex and thus may be best understood using mixed methods. Only a few studies combined quantitative surveys with qualitative interviews or FGDs to triangulate findings to gain a comprehensive understanding of the studied behaviors. Second, we found little consistency in measurement approaches across studies even when assessing the same behavior. For instance, when assessing purchase frequency of organic foods using 5-point Likert scales, 1 study asked about how often respondents shopped for organic foods [15], whereas another study asked respondents’ level of agreement to a statement about occasionally purchasing organic foods [16], thus making it challenging to compare findings between these 2 studies. Third, there was a lack of studies using validated tools (*n* = 14), despite our inclusive definition of a validated tool, i.e., authors reporting that the tool was validated. Interestingly, 13 of the 14 validated tools were used to assess these behaviors in youth, and all but 1 tool was validated in India. This underscores a geographic and demographic limitation of the current validated tools. Although the validated tools covered a range of behaviors (school food, fast food, meat, and organic food purchasing behaviors, general eating habits, cravings, meal skipping, snacking, eating out, and fast food consumption), their limited scope and contextual specificity diminish their broader applicability. Developing and implementing new tools to assess food choice behaviors is a time- and resource-consuming exercise. However, the continued reliance on tools that have not been validated, especially those not tested across multiple settings, limits the utility, comparability, and generalizability of findings. Although we recognize that the inherent heterogeneity of food choice behaviors in different contexts necessitates a variety of measurement approaches, tools that have not been demonstrated to measure what they intend to measure have limited utility. Future research should prioritize the development, validation, and harmonization of tools that can be applied across diverse populations and settings. Utilization of existing validated tools can provide useful data for comparison across populations and contexts and over time to assess trends in food choice behavior.

### Evidence gaps

This review highlighted some gaps in evidence on the 3 food choice behaviors examined. Most studies in our review examined food acquisition (*n* = 26 studies) and household consumption practices (*n* = 30), whereas food preparation behaviors were relatively understudied (*n* = 9). It is important to study these behaviors in varied contexts. However, most of the reviewed studies were based in India (*n* = 37) and in urban settings (*n* = 25). There was a lack of evidence from rural (*n* = 3) and periurban (*n* = 1) settings. Studies focusing on younger children (*n* = 2) and adolescents (*n* = 8) were uncommon.

Quantitative tools were commonly used to examine food acquisition behaviors (*n* = 18) and household consumption practices (*n* = 18), whereas qualitative tools were commonly used to examine food preparation behaviors (*n* = 6). Most studies that examined food acquisition behaviors focused on specific foods, especially organic foods. Understanding acquisition behaviors at the food-group level, such as fruits and vegetables [34], is relevant for analyzing possible variation in diets and supporting policies that promote healthier food choices.

### Strengths and limitations

The key strength of this study is that it provides a review of tools used to assess a diverse set of behaviors—food acquisition, food preparation, and household consumption practices—in 8 countries. A varied set of studies examining different behaviors was included. For example, several studies considered specific foods, such as nonpackaged nonbranded rice, unhealthy snacks, probiotic food and beverages, and halal food, rather than general food choices. Additionally, some studies examined food purchasing behaviors during the COVID-19 pandemic. Although these studies may not be reflective of usual practices, they provide a breadth of evidence on measuring food choice behavior. The primary limitation of the study is that evidence may have been unaccounted for because of search exclusions such as exclusion of gray literature or non-English articles. Our review did not include food storage and disposal behaviors, which could be important for their contribution to the study of food waste and sustainability practices. Additionally, we did not extract information about the funding source for each included study, which could have played a role in determining the type of food choice behavior that was examined in each study.

### Moving toward better measurement of food choice behaviors

Our review highlights that although using various tools, often tailored to specific behaviors, is valuable for capturing the complexity of food choice behaviors, inconsistent approaches to measuring the same elements across studies create significant heterogeneity, limiting opportunities for comparability and cumulative understanding. With multiple tools to measure the same element, many tools lack thorough development, validation, and accessibility, further complicating high-quality and reproducible measurements. Not reporting the complete set of tools used hinders researchers from reproducing, adapting, and improving existing methods. There is a need for greater standardization of assessment tools across contexts and clear documentation of the methods and instruments used. Academic journals should require the inclusion of assessment tools, and peer reviewers should be invited to scrutinize the tools. A publicly accessible repository of tools that can be adapted to different contexts, ideally with complete information such as steps taken to validate each tool as well as where and for whom each tool has been used, would be a valuable contribution to the scientific community. Efforts have started to develop a centralized repository of existing measures, instruments, and protocols to assess food choice behaviors, but this work remains incomplete and requires further refinement to maximize its utility [8]. Adapting and validating existing tools, rather than creating new ones, can improve efficiency, continuity, and comparability, enabling researchers to focus on advancing our understanding of food choice behaviors. Moving forward, there is a critical need to prioritize the harmonization and validation of tools, especially through testing across diverse populations and settings. This will allow for more meaningful comparisons, enable progress tracking over time across regions, and improve the evidence base for designing effective interventions and policies. Addressing these gaps in food-related behaviors is critical for designing culturally sensitive, contextually appropriate strategies aimed at improving dietary practices and nutritional outcomes.

## Author contributions

The authors’ responsibilities were as follows – CEB, RA, SC, MB, SP: designed the review protocol; SP, MB: conducted the search and extracted the data; SS, RA, CEB: independently reviewed the extracted data and resolved any disagreements; FA, JKD, RS, PM: aided the manuscript writing process; SP: had primary responsibility for final content; and all authors: read and approved the final manuscript.

## Data availability

Data described in the manuscript are available as a supplemental spreadsheet.

## Funding

Funding for this work was provided by the CGIAR Trust Fund through the Transforming Agrifood Systems in South Asia initiative.

## Conflict of interest

PM reports financial support was provided by CGIAR Trust Fund. If there are other authors, they declare that they have no known competing financial interests or personal relationships that could have appeared to influence the work reported in this paper.
