# Supplementary material for: Food Acquisition, Preparation, and Consumption Practices in South Asia: A Scoping Review of Assessment Tools
Source: Adv Nutr. 2025 Sep 18;16(11):100518. doi: 10.1016/j.advnut.2025.100518 (PMC12547306; doi:10.1016/j.advnut.2025.100518)
Supplement: Multimedia component 2 [file mmc2.docx]

**Supplemental Table 1:** Search strings used after initial scoping.

|  | Keyword |
| --- | --- |
| Food acquisition | (“food purchas*” OR “food buy*” OR “food shop*” OR “food acqui*” OR “food vendor*” OR “food”  AND  (“famil*” OR “home” OR “household*” OR “individual*”)  AND  (“barter*” OR “exchang*” OR “trad*” OR “grow*” OR “produc*”))  AND  (“Afghanistan” OR “Bangladesh” OR “Bhutan” OR “India” OR “Maldives” OR “Nepal” OR “Sri Lanka” OR “Pakistan”) |
| Food preparation | (“cook*” OR “food prepar*” OR “meal prepar*” OR “meal plan” OR “meal plans” OR “meal planning” OR “home prepar*” OR “homemade” OR ((“food” OR “meal*” OR “ingredient” OR “cook*”)  AND  (“bake” OR “baking” OR “fry” OR “frying” OR “clean*” OR “chop*” OR “boil*” OR “broil*” OR “grill*”)))  AND  (“Afghanistan” OR “Bangladesh” OR “Bhutan” OR “India” OR “Maldives” OR “Nepal” OR “Sri Lanka” OR “Pakistan”) |
| Household eating patterns | (“eating episode” OR “food consumption behavior” OR ((“meal*” OR “breakfast” OR “lunch” OR “dinner” OR “dessert” OR “snack*” OR (“consum*” AND “food”) OR “meal*” OR “eat” OR “eating”)  AND  (“pattern*” OR “sequence” OR “speed” OR “location” OR “skip*” OR “on-the-go” OR “take out” OR “away from home” OR “home*” OR “travel*” OR “share” OR “shares” OR “sharing” OR “shared” OR “separate” OR “sit” OR “sitting” OR “fami*” OR “time” OR “timing” OR “episode” OR “occasion” OR “synchronicity” OR “event*” OR “moment” OR “periodicity” OR “tempo” OR “synchronization” OR “synchronsation” OR “frequency” OR “famil*”)))  AND  (“Afghanistan” OR “Bangladesh” OR “Bhutan” OR “India” OR “Maldives” OR “Nepal” OR “Sri Lanka” OR “Pakistan”) NOT (“minimum meal frequency” OR “food frequency questionnaire”) |

| **Supplemental Table 2:** Geographical, population, and methodological characteristics of included studies | | | | | |
| --- | --- | --- | --- | --- | --- |
| **Study reference** | **Country** | **Study population** | **Data collection methods** | **Food choice behavior(s) assessed** | **Domains of food choice driver(s) assessed** |
| Aggarwal et al. 2006 | India | Adolescents (n=500 females and 500 males) | Interviewer-administered questionnaire | Household consumption practices | Intrapersonal, socio-cultural, personal food environment |
| Ali et al. 2021 | India | Adults (n=66 females and 122 males) | Interviewer-administered questionnaire | Food acquisition | Intrapersonal, personal food environment, material assets and resources |
| Arora et al. 2021 | India | Adults (n=102 females and 204 males) | Interviewer-administered questionnaire | Food acquisition | Intrapersonal, socio-cultural, personal food environment |
| Ashraf 2019 | Bangladesh | Adolescents and adults (n=37 females and 63 males) | Interviewer-administered questionnaire | Food acquisition | Intrapersonal, material assets and resources |
| Bailey et al. 2018 | India | Adults (n=38 females) | Semi-structured interviews, pile sorting | Food preparation, Household consumption practices | Intrapersonal, socio-cultural, personal food environment, person-state |
| Bhol et al. 2021 | India | Adults (n=196 females and 60 males) | Self-administered questionnaire | Household consumption practices | Socio-cultural, person-state |
| Blum et al. 2019 | Bangladesh | Adolescents (n=24) | Semi-structured interviews | Household consumption practices | Socio-cultural, personal food environment |
| Budhathoki, M., & Pandey, S. 2021 | Nepal | Adults (n=304 females and 224 males) | Interviewer-administered questionnaire | Food acquisition | Intrapersonal, socio-cultural, personal food environment |
| Bukhari et al. 2022 | Pakistan | Adults (n=46 females and 44 males) | Semi-structured interviews | Food acquisition | Socio-cultural, personal food environment, person-state |
| Chakrabarti, S. 2010 | India | Adults (n=33 experts) | Interviewer-administered questionnaire | Food acquisition | Intrapersonal, socio-cultural, personal food environment |
| Chopra et al. 2021 | India | Adolescents (n=20 females and 16 males) and adult caregivers (n=23 females) | Focus group discussions (FGDs) | Food acquisition, Food preparation, Household consumption practices | Intrapersonal, socio-cultural, personal food environment |
| Dahal et al. 2022 | Nepal | Adolescent and adult students (n=194 females and 191 males) | Self-administered questionnaire | Household consumption practices | Intrapersonal, socio-cultural, personal food environment |
| Das et al. 2021 | India | Adults (n=68 females and 82 males) | Self-administered questionnaire, semi-structured interviews | Food acquisition, Food preparation | Intrapersonal, person-state |
| David et al. 2020 | India | Adults (n=112 females and 128 males) | Interviewer-administered questionnaire | Food acquisition | Intrapersonal |
| Ganpule et al. 2023 | India | Adults (n=4949 females and 3813 males) | Interviewer-administered questionnaire | Household consumption practices | Intrapersonal, material assets and resources |
| Ghatak et al. 2023 | India | Adults (n=1125 households each with at least one female and one male aged up to 18 years) | Interviewer-administered questionnaire, photovoice | Household consumption practices | Socio-cultural, material assets and resources |
| Kabir et al. 2018 | Bangladesh | Adolescents and adults (Interviews: n=9 females and 19 males, FGDs: n=10 females and 16 males) | Semi-structured interviews, focus group discussions | Food preparation, Household consumption practices | Intrapersonal, socio-cultural, material assets and resources |
| Kaur et al. 2020 | India | Adults (n=30 females and 23 males) | Focus group discussions | Household consumption practices | Intrapersonal, personal food environment |
| Khan et al. 2021 | India | Adult consumers (n=54 females and 103 males) | Interviewer-administered questionnaire | Household consumption practices | Intrapersonal |
| Khanna et al. 2022 | India | Adult consumers (n=240 females and 205 males) | Self-administered questionnaire | Household consumption practices | Intrapersonal, socio-cultural, personal food environment, person-state |
| Khongrangjem et al. 2018 | India | Adolescent and adult Pre-University College students (n=77 females and 83 males) | Self-administered questionnaire | Household consumption practices | Intrapersonal |
| Kiran et al. 2018 | India | Adolescents and adults (n=66 females and 194 males) | Interviewer-administered questionnaire | Food acquisition | Intrapersonal, personal food environment |
| Kumar et al. 2017 | India | School-going adolescents (n=885 females and 767 males) | Self-administered questionnaire | Household consumption practices | Intrapersonal |
| Malushte et al. 2022 | India | Adolescents (n=96 females and 104 males) | Interviewer-administered questionnaire | Food acquisition | Intrapersonal, socio-cultural, personal food environment |
| Matharu et al. 2022 | India | Adult higher education students (n=186 females and 215 males) | Self-administered questionnaire | Food acquisition | Intrapersonal, socio-cultural, personal food environment |
| Matharu et al. 2023 | India | Adult higher education students (n=186 females and 215 males) | Self-administered questionnaire | Food acquisition | Intrapersonal, personal food environment |
| Menon et al. 2022 | India | Adult female food gatekeepers/ those responsible for feeding their family members (n=34) | Semi-structured interviews | Food acquisition, Food preparation, Household consumption practices | Socio-cultural |
| Mohanty et al. 2017 | India | Adult medical students (n=126 females and 152 males) | Interviewer-administered questionnaire | Household consumption practices | Intrapersonal, personal food environment |
| Moitra, P. & Madan, J. 2022 | India | Adolescents (n=343 females and 369 males) | Self-administered questionnaire | Food acquisition, Household consumption practices | Intrapersonal, personal food environment |
| Mor, K., & Sethia, S. 2018 | India | Adults (n=1421) | Interviewer-administered questionnaire | Household consumption practices | Intrapersonal, personal food environment |
| Neogy, S. 2010 | India | Adults (families in the community) | Focus group discussions, pile sorting | Household consumption practices | Intrapersonal, socio-cultural |
| Patel et al. 2010 | India | Adolescents (n=101 females and 50 males) | Interviewer-administered questionnaire | Household consumption practices | Intrapersonal, socio-cultural, personal food environment |
| Pradhan et al. 2013 | India | Adults (20 households and 4 key informants) | Interviewer-administered questionnaire, semi-structured interviews | Food acquisition, Household consumption practices | Intrapersonal |
| Prasad, R., & Umesh, S. 2016 | India | Not specified (n=171 respondents) | Self-administered questionnaire | Food acquisition | Intrapersonal, personal food environment |
| Qazi et al. 2022 | Pakistan | Adult students (n=36 females and 84 males) | Semi-structured interviews | Food acquisition | Socio-cultural, personal food environment, person-state |
| Rahman et al. 2021 | India | Not specified (n=416 respondents) | Self-administered questionnaire | Food acquisition | Intrapersonal, personal food environment |
| Renzella et al. 2020 | Sri Lanka | Adults (n=62 females and 32 males) | Semi-structured interviews | Food preparation | Intrapersonal, socio-cultural, material assets and resources |
| Roy et al. 2021 | India | Younger children, adolescents, and adults (n=2355 females and 2254 males) | Semi-structured interviews | Household consumption practices | Intrapersonal, personal food environment, material assets and resources, person-state |
| Samanta et al. 2022 | India | Adults (n=741 females and 318 males) | Self-administered questionnaire | Food acquisition, Food preparation | Intrapersonal, socio-cultural, person-state |
| Semar, R., & Bakshi, N. 2022 | India | School-going younger children (n=73 females and 27 males) | Self-administered questionnaire | Household consumption practices | Intrapersonal, personal food environment, person-state |
| Shahzad et al. 2022 | Pakistan | Adults (n=430 females and 637 males) | Self-administered questionnaire, photovoice | Food acquisition | Socio-cultural |
| Turner et al. 2022 | India | Adults (n=16 females and 20 males) | Semi-structured interviews, photovoice | Food acquisition, Food preparation, Household consumption practices | Personal food environment |
| Ullah et al. 2023 | Pakistan | Adults (46 females and 44 males) | Semi-structured interviews | Food acquisition | Intrapersonal, socio-cultural, personal food environment |
| Verma et al. 2023 | India | Adolescents and adults (n=351 female, 371 male children and their parents) | Interviewer-administered questionnaire | Household consumption practices | Intrapersonal, socio-cultural |
| Vinish et al. 2021 | India | Adults (n=385) | Self-administered questionnaire, Interviewer-administered questionnaire | Food acquisition | Intrapersonal, personal food environment, |
| Ynion et al. 2021 | India | Adults (n=501) | Interviewer-administered questionnaire, focus group discussions | Food acquisition, Household consumption practices | Intrapersonal, personal food environment |
